# Terminal fucosylation of haptoglobin in cancer-derived exosomes during cholangiocarcinoma progression

**DOI:** 10.3389/fonc.2023.1183442

**Published:** 2023-04-24

**Authors:** Hyewon Choi, Sungeun Ju, Keunsoo Kang, Moon-Hyeong Seo, Jin-Man Kim, Eiji Miyoshi, Min-Kyung Yeo, Seung-Yeol Park

**Affiliations:** ^1^Department of Life Sciences, Pohang University of Science and Technology (POSTECH), Pohang, Gyeongbuk, Republic of Korea; ^2^Department of Microbiology, Dankook University, Cheonan, Chungnam, Republic of Korea; ^3^Natural Product Research Center, Korea Institute of Science and Technology, Gangneung, Republic of Korea; ^4^Department of Pathology, Chungnam National University School of Medicine, Daejeon, Republic of Korea; ^5^Department of Molecular Biochemistry and Clinical Investigation, Osaka University Graduate School of Medicine, Osaka, Japan

**Keywords:** cholangiocarcinoma (CCA), glycosylation, haptoglobin (Hp), exosome, biomarker

## Abstract

**Background:**

Cholangiocarcinoma (CCA) is a silent tumor with a high mortality rate due to the difficulty of early diagnosis and prediction of recurrence even after timely surgery. Serologic cancer biomarkers have been used in clinical practice, but their low specificity and sensitivity have been problematic. In this study, we aimed to identify CCA-specific glycan epitopes that can be used for diagnosis and to elucidate the mechanisms by which glycosylation is altered with tumor progression.

**Methods:**

The serum of patients with various cancers was fractioned into membrane-bound and soluble components using serial ultracentrifugation. Lectin blotting was conducted to evaluate glycosylation. Proteins having altered glycosylation were identified using proteomic analysis and further confirmed using immunoblotting analysis. We performed HPLC, gene analysis, real-time cargo tracking, and immunohistochemistry to determine the origin of CCA glycosylation and its underlying mechanisms. Extracellular vesicles (EV) were isolated from the sera of 62 patients with CCA at different clinical stages and inflammatory conditions and used for glycan analysis to assess their clinical significance.

**Results:**

The results reveal that glycosylation patterns between soluble and membrane-bound fractions differ significantly even when obtained from the same donor. Notably, glycans with α1-3/4 fucose and β1-6GlcNAc branched structures increase specifically in membrane-bound fractions of CCA. Mechanically, it is primarily due to β-haptoglobin (β-Hp) originating from CCA expressing fucosyltransferase-3/4 (FUT 3/4) and N-acetylglucosaminyltransferase-V (MGAT5). Newly synthesized β-Hp is loaded into EVs in early endosomes *via* a KFERQ-like motif and then secreted from CCA cells to induce tumor progression. In contrast, β-Hp expressed by hepatocytes is secreted in a soluble form that does not affect CCA progression. Moreover, evaluation of EV glycosylation in CCA patients shows that fucosylation level of EV-Hp gradually increases with tumor progression and decreases markedly when the tumors are eliminated by surgery.

**Conclusion:**

This study suggests that terminal fucosylation of Hp in cancer-derived exosomes can be a novel glycan marker for diagnosis and prognosis of CCA. These findings highlight the potential of glycan analysis in different fractions of serum for biomarker discover for other diseases. Further research is needed to understand the role of fucosylated EVs on CCA progression.

## 1 Introduction

CCA is a malignant epithelial tumor arising from the intrahepatic and extrahepatic bile ducts with a five-year survival rate of 10%, accounting for 13% of total cancer-related mortality (1, 2). CCA is diagnosed based on a combination of clinical, radiological, serological, and histological findings. However, differentiating benign from malignant biliary strictures during early diagnosis is challenging because CCA is often confused with a wide spectrum of inflammatory conditions within the bile ducts (3). General biomarkers, including carcinoembryonic antigen (CEA), CA19‐9, and CA125, are widely used for diagnosis, but there are issues of low sensitivity and specificity (4, 5). Therefore, it is necessary to develop non-invasive biomarkers for diagnosing CCA at an early stage and predicting prognosis.

Glycosylation is a major post-translational modification that plays a pivotal role in the function and stability of proteins, lipids, and nucleotides (6–8). The discovery of aberrant glycosylations in cancers has highlighted their potential for diagnosis (6, 7). β-Hp is an acute-phase glycoprotein mainly secreted by hepatocytes and changes glycosylation status in various cancers (9). In prostate cancer, β-Hp exhibits an increased amount of glycans with β1-6GlcNAc side chain and α1-3/4 fucose (10, 11). Among the four N-glycosylation sites in β-Hp (Asn 184, 207, 211, and 241), fucosylated glycans were specifically increased at Asn 211 in prostate and pancreatic cancer, as opposed to that at Asn 241 in colorectal cancer (10, 12, 13). How β-Hp glycosylation is regulated in cancers has been investigated. Okuyama et al. uncovered that pancreatic cells secrete IL-6, which induced expression of fucosylation-regulatory in hepatocytes to produce α1-6 fucosylated β-Hp (14). We previously found that α1-3/4 fucosylated β-Hp is expressed in colon cancer cells (13, 15). However, the molecular understanding of how β-Hp with aberrant glycosylation is secreted from different cells remains unclear.

Proteins can be secreted from cells in two forms: (i) soluble forms and (ii) membrane-bound forms involving EV. EVs are nano-sized vesicles and produced in the multivesicular body (MVB) or plasma membrane by the endosomal sorting complex required for transport (ESCRT) complex and Rab27 GTPase (16, 17). Since EVs carry several factors implicated in long-distance intercellular communication, tumor progression, organotropic metastasis, and immune suppression (18–20), it has been considered as potential biomarkers and therapeutic carriers. How proteins are secreted in a soluble form was extensively studied, but the mechanism by which they are secreted through EV remains unclear (21, 22). Cytosolic cargoes are sorted into the EV during vesicle formation through binding to ubiquitinated adaptor proteins and ESCRT complex (23, 24). Conversely, secretory molecules bind to LAMP2A *via* KFERQ motif and are then sorted into EVs in the early endosome (25, 26). Recent studies uncovered that many cargoes, previously known to be secreted in a membrane-free form, can also be secreted through EV (27). However, how the two forms differ needs to be determined.

In the present study, we initially performed lectin-based glycan analysis on different fractions of sera from patients with various solid cancers and found that α1-3/4 fucosylation in a branched glycan structure was explicitly increased in membrane fraction of CCA. The molecular mechanisms of how CCA-specific glycosylation is regulated were elucidated. Moreover, the clinical significance of EV glycosylation in cancer diagnosis and prognosis was investigated. Our results provide novel insights into how cancer glycosylation is regulated, along with their significance as a biomarker for CCA.

## 2 Materials and methods

### 2.1 Patients and sample collection

For this retrospective study, we enrolled patients previously diagnosed at Chungnam National University Hospital (Daejeon, Korea) between January 2009 and March 2021: 87 patients with solid cancer who underwent surgical treatment, 20 with inflammatory biliary disease, and 28 healthy volunteers. The solid cancers included cholangiocarcinoma (CCA, n=42), hepatocellular carcinoma (HC, n=10), lung adenocarcinoma (LC, n=10), gastric adenocarcinoma (GC, n=10), breast ductal carcinoma (BC, n=5), and prostatic adenocarcinoma (PC, n=10) at various clinical stages. Of the 42 patients with CCA, 31 had extrahepatic cholangiocarcinoma (EHCC) and 11 had intrahepatic cholangiocarcinoma (IHCC). Patients were diagnosed with stage I (n=8), II (n=27), III (n=6), or IV (n=1) disease. Pre-operative blood samples (within a week before the operation) from patients with solid cancer were collected during the first surgery. During preoperative serum sample collection, none of the patients received preoperative chemotherapy or radiotherapy. Patients with stage II tumors were administered adjuvant gemcitabine and cisplatin chemotherapy. Follow-up blood samples were collected from 10 patients during recurrence evaluation after chemotherapy. Forty-two primary CCA tissue samples were obtained in the form of formalin-fixed, paraffin-embedded tissue blocks. The inflammatory biliary diseases included cholangitis (n=15) and cholecystitis (n=5). This study was approved by the Institutional Review Board of Chungnam National University Hospital (IRB file no. CNUH 2020-09-015).

### 2.2 Cells and plasmids

Cholangiocarcinoma cell lines, SNU-1079, was cultured in completed RPMI1640 (Welgene) and HepG2 in completed MEM with EBSS (cytiva) at 37°C and 5% CO2. Transfection of DNA plasmids was performed using Genjet (SignaGenLab). The custom plasmid encoding GFP-tagged haptoglobin was obtained from VectorBuilder. The mutant form of haptoglobin was generated using QuickChange II Site-Directed Mutagenesis (Agilent). For retention using the selective hooks (RUSH) system, GFP-Hp was subcloned into pIRESneo3 using PCR. To examine cell growth upon treatment with EV and soluble Hp, SNU-1079 cells (1 × 10^5^) in 24-well plates were treated with EVs (100 μg/ml) or soluble Hp (0.2 μg/ml), followed by counting the cell number every 1 day.

### 2.3 Antibodies, lectins, and chemicals

The antibodies used were rabbit anti-giantin antibody (ab80864), rabbit anti-Rab11A antibody (ab128913), and rabbit anti-CD9 antibody (ab92726) from Abcam; rabbit anti-Rab5 antibody (3547) from Cell Signaling Technology; mouse anti-Alix antibody (sc-53540) from Santa Cruz Biotechnology; mouse anti-CD63 antibody (556019) from BD Biosciences; rabbit anti-TSG101 antibody (102286-T38) from Sino Biological; polyclonal rabbit antibody against haptoglobin (H8636) from Sigma Aldrich; polyclonal rabbit antibody against FUT3 (DF4068) from Affinity Biosciences; rabbit antibody against FUT4 (A16320) from ABclonal; HRP-conjugated goat anti-mouse IgG (H+L) (145553), HRP-conjugated goat anti-rabbit IgG (H+L) (145164), Cy3-conjugated goat anti-mouse IgG (H+L) (144931), and Alexa Fluor 647-conjugated goat anti-rabbit IgG (H+L) (145016) from Jackson ImmunoResearch Laboratories. HRP-conjugated streptavidin (21126) was obtained from Thermo Scientific. The following lectins were purchased from Vector Laboratories: biotinylated Sambucus nigra lectin (SNA, B-1305), biotinylated Phaseolus vulgaris leucoagglutinin (PHAL, B-1115), biotinylated Aleuria aurantia lectin (AAL, B-1395), and biotinylated wheat germ agglutinin (WGA, B-1025). Flavobacterium meningosepticum PNGase F (P0704S), Arthrobacter ureafaciens α2-3,6,8,9 Neuraminidase A (P0722S), Streptococcus pneumoniae β1-4 galactosidase S (P0745S), and Xanthomonas manihotis β-N-acetylglucosaminidase S (P0744S) were purchased from New England Biolabs. X. manihotis α1-3,4 fucosidase (E-F134) was obtained from QA-bio.

### 2.4 Glycan analysis of different fractions in serum using various lectins

To isolate soluble components and membrane-bound components from serum, we employed serial centrifugation. 500 μL of serum was diluted five times with PBS and then centrifuged at 2,000 × g for 30 min at 4°C. The supernatant was subjected to ultracentrifugation at 200,000 × g for 2 h at 4°C. Soluble components were collected from the supernatant and the membrane-bound components were obtained from the pellet fraction. Glycosylation profiles were determined *via* lectin blotting (15). Briefly, isolated soluble components and membrane-bound components (2–10 μg) were subjected to SDS-PAGE, followed by transfer to a PVDF membrane (Millipore). After blocking with 3% BSA, the membrane was incubated with biotinylated lectins for 18 h at 4°C. The following lectins were used: PHAL (GlcNAcβ1-6Manα1-6Man side chain; 1:500 dilution), SNA (NeuAcα2-3/6Gal/GalNAc; 1:1000), WGA (GlcNAc and NeuAc; 1:500), and AAL (Fucα1-3/4/6GlcNAc; 1:1000). The membrane was incubated with HRP-conjugated streptavidin (1:10000) in TBS-T+ (20 mM Tris pH 7.4, 150 mM NaCl, 0.9 mM CaCl2, 0.5 mM MgSO4, and 0.1 mM MnCl2, and 0.5% Tween-20) for 30 min and then developed using ECL solution (Enzynomics). For reblotting, the membrane was incubated with stripping buffer (LPS solution) for 1 h, blocked with 5% skim milk/TBS-T, and then incubated with an anti-haptoglobin antibody (1:2000). The membrane was then incubated with goat anti-rabbit IgG-HRP (1:5000) and developed using ECL solution. The reactivity of lectins was quantified using Image J.

### 2.5 Isolation and characterization of EV

We employed serial centrifugation or Exoquick (EXOTC10A-1, System Biosciences) to isolate EVs from the patient serum as previously described (28). To isolate EV using serial centrifugation (SC), 500 μl of serum centrifuged at 2,000 × g for 30 min at 4°C. The supernatant was subjected to ultracentrifugation at 200,000 × g for 2 h at 4°C. EV was obtained from the pellet fraction. To isolate EV using Exoquick (EQ), 500 μl of serum centrifuged at 3000 × g for 30min at 4°C. The supernatant was mixed with 200 ul of Exoquick, followed by incubation for 18hr at 4°C and centrifuged at 1500 × g for 30 min at 4°C. EV was obtained from the pellet fraction. To isolate EV from cell cultured media, we employed serial centrifugation. Cells were cultured to 80% confluence in a serum-free medium for 2 days. The culture medium centrifuged at 2,000 × g for 30 min at 4°C. The supernatant was subjected to ultracentrifugation at 175,000 × g for 2 h at 4°C. EVs were obtained from the pellet. Isolated EVs were characterized using western blot with antibodies against TSG101, Alix, and CD9, nanoparticle tracking analysis, and electron microscopy. Glycosylation profiles of EV were determined *via* lectin blotting using PHAL, SNA, WGA and AAL.

### 2.6 Purification of β-haptoglobin from serum using immune-affinity chromatography

β-Hp was purified from soluble and membrane-bound fraction of serum using immunoaffinity chromatography, as previously described (15). Briefly, soluble fraction (1 mL) and membrane-bound fraction (5 mg) were loaded onto an anti-Hp antibody-conjugated sepharose 4 B column (2 mL). The column was washed with 25 mL of PBS at 1 mL/min using a peristaltic pump. β-Hp was eluted using 6 mL elution buffer (0.1M Glycine, 0.5M NaCl, pH 2.8) and neutralized with 250 μL of neutralization buffer (1.0M Tris-HCl, pH 9.0). The eluent was concentrated using a centrifugal filter (MWCO 10,000; Amicon Ultra; Millipore). Purified β-Hp was quantified using a Bradford assay and stored at -80°C until use.

### 2.7 Detailed examination of N-glycan structures using HILIC-HPLC

N-Glycosylation structures on isolated EVs were characterized using a hydrophilic interaction chromatography (HILIC)-HPLC system (15). N-Glycans of EV-Hp were released by treatment with PNGaseF using in-gel digestion and then labeled with 2-aminobenzamide (2-AB) using a LudgerTag 2AB Glycan Labeling Kit (Ludger). The 2-AB-labeled glycans were separated from free 2-AB using LudgerClean S cartridges (Ludger), separated *via* HPLC through Xbridge Glycan BEH Amide XP 2.5 μm, 3.0 mm × 150 mm column (Waters, Milford, MA, USA) using the e2695 separation module, and finally detected using a 2475 fluorescence detector. Chromatography was performed at 60°C. The mobile phase consisted of Sol A (25%, v/v, 50 mM ammonium formate pH 4.4 (Sigma Aldrich) and Sol B (75%, v/v, acetonitrile), followed by a linear gradient of Sol A from 25% to 45% over 65 min at a flow rate 0.56 mL/min. The retention time of N-glycans was compared with that of the 2-AB dextran ladder. For exoglycosidase treatment, 2-AB-labeled glycans were incubated with different combinations of exoglycosidases in 50 mM sodium acetate buffer (pH 5.5) at 37°C for 24 h and then subjected to HILIC-HPLC.

### 2.8 *In vivo* transport assays

We employed a quantitative assay that tracks protein transport in cells using a RUSH system (29). Briefly, cells were transfected with GFP-Hp in pIRESneo3 to synchronize β-Hp conjugated with streptavidin-binding peptide in the ER through interaction of the ER-localized “hook” with streptavidin. The cells were then treated with 40 nM D-biotin (B4501, Sigma Aldrich) to release β-Hp by competing with streptavidin for SBP binding, and fixed at different time points using 4% PFA/PBS for 10 min and stained using antibodies against giantin, Rab5, Rab11, and CD63. The cells were imaged using a Zeiss LSM800 confocal microscope with a Plan-Apochromat 63x objective, Zeiss URGB (488, 561, and 647 nm) laser lines, and Zen 2.3 confocal acquisition software. Images were merged using Image J and analyzed using MetaMorph 7.7 (MDS Analytical Technologies).

### 2.9 Immunohistochemistry

Immunohistochemical analysis was performed on samples from 42 patients. Whole FFPE tissues were sectioned on coated slides, deparaffinized with xylene, and hydrated in serial solutions of alcohol. The sections were heated for 3 min in a pressure cooker (containing 10 mM sodium citrate, pH 6.0) for antigen retrieval. Endogenous peroxidase was blocked using 0.03% hydrogen peroxide for 10 min. The sections were incubated at room temperature for 1 h with the following primary antibodies: FUT3 (1:200), FUT4 (1:200), TSG101 (1:200), CD63 (1:200), and haptoglobin (1:400). Hepatocytes in liver FFPE samples were used as positive controls, and tonsil tissues were used as negative controls. The staining intensities were designated as negative, weak, moderate, or strong, and the staining proportions were determined in increments of 5% across a 0–100% range. The histo-score (H-score), a summation of the proportion of area stained at each intensity level multiplied by the weighted staining intensity (e.g., 0, negative; 1, weak; 2, moderate; 3, strong), was determined for the sections (30).

### 2.10 Statistical analysis

Data are presented as mean ± s.e.m. Biological repeat counts are indicated in the figure legend. Statistical analysis was performed using GraphPad Prism (v. 9) using an unpaired two-tailed Student’s t-test for data with homogeneity of variance. A Mann-Whitney u-test was performed on data with heteroscedasticity of variance. A P-value of less than 0.05 was considered statistically significant.

## 3 Results

### 3.1 Glycan analysis on different fractions in the serum of cancer patients

**Figure 1:**
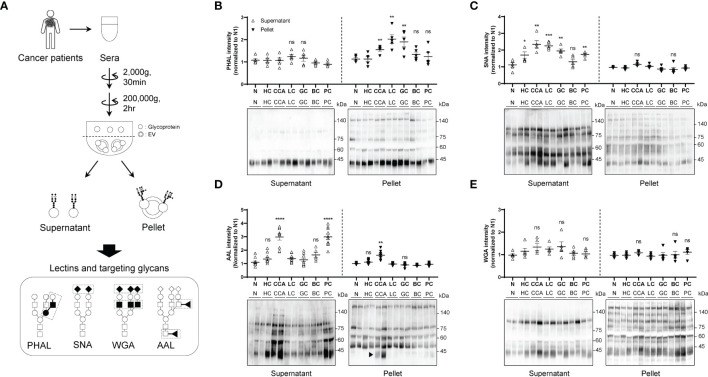
Comparison of glycosylation between soluble and membrane-bound fraction in the serum of different cancers. Quantitative data are shown as mean ± S.E.M. Statistical analysis was performed using the two-tailed Student’s t-test. ****p < 0.0001, ***p < 0.001, **p < 0.01, *p < 0.05, ns p > 0.05. **(A)** Schematic illustration of glycosylation comparison between soluble fraction and membrane-bound fraction. ◼: GlcNAc, ●: mannose, ○: galactose, ▲: fucose, ◆: sialic acid. **(B)** PHAL blotting for different fractions in the sera from different cancers; n = 5. CCA, cholangiocarcinoma; HC, hepatocellular carcinoma; LC, lung adenocarcinoma; GC, gastric adenocarcinoma; BC, breast ductal carcinoma; PC, prostatic adenocarcinoma. **(C)** SNA blotting for different fractions in the sera from different cancers; n = 5. **(D)** AAL blotting for different fractions in the sera from different cancers; n = 5. **(E)** WGA blotting for different fractions in the sera from different cancers; n = 5. ns, not significant.

To investigate whether the glycosylation status differs between soluble and membrane-bound fractions in the blood, we used serial centrifugation to fractionate the sera of patients with various cancers (CCA, HC, LC, GC, BC, and PC) (**Figure 1A**). We then analyzed the glycosylation patterns using various lectins (**Figure 1A**). We found that the membrane-bound components of CCA, LC and GC contained an increased amount of glycans with β1-6GlcNAc branching structure, which was not changed in the soluble components between samples (**Figure 1B**). SNA blotting revealed that most cancers contained enhanced sialylation in the soluble components compared to controls (**Figure 1C**). However, we did not observe any significant difference in the level of sialylation in the membrane-bound fraction between samples (**Figure 1C**). Additionally, AAL blotting showed that the soluble components of CCA and PC contained higher levels of fucosylation than other cancers and healthy controls (**Figure 1D**). Interestingly, the membrane-bound components of CCA showed a specific increase in fucosylation, with the AAL signal predominantly increasing at 40 kDa (**Figure 1D**; arrowhead). However, we did not find significant difference in the glycans detected by WGA between the different fractions and cancer types (**Figure 1E**).

### 3.2 Aberrant glycosylation in the sera of CCA is attributed to membrane-bound β-Hp

**Figure 2:**
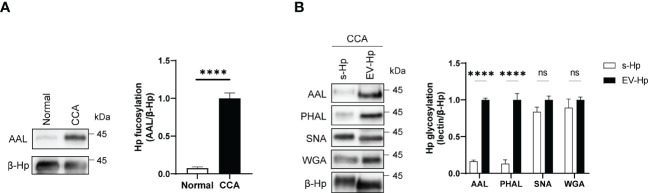
Glycosylation of EV-Hp is correlated with CCA. Quantitative data are shown as mean ± S.E.M. Statistical analysis was performed using the two-tailed Student’s t-test. ****p < 0.0001, ns p > 0.05. **(A)** AAL blotting for β-Hp purified from EVs; n = 6. **(B)** Lectin blotting for s-Hp and EV-Hp; n = 3. ns, not significant.

We next conducted proteomic analysis and identified β-Hp as the glycoprotein with increased fucosylation in the membrane-bound components of CCA (**Supplementary Figure 1A**). We then purified β-Hp from membrane-bound fractions using immune-affinity chromatography and confirmed that β-Hp from CCA exhibited higher fucosylation than that from normal (**Figure 2A**). Given that the amounts of β-Hp were comparable between CCA and normal (**Supplementary Figure 1B**), the increased fucosylation was attributed to the glycosylation status, rather than changes in protein amount. We then noted that β-Hp exists in the blood in two forms: soluble forms (s-Hp) and membrane-bound forms associated with EV (EV-Hp) (31–33). To determine which form is more correlated with CCA, we purified β-Hp from soluble and membrane-bound fractions in the sera of the same patient. We found that EV-Hp showed higher reactivity to AAL and PHAL than s-Hp, whereas both forms of β-Hps showed similar signal intensities for WGA and SNA (**Figure 2B**). These results suggest that the aberrant glycosylation observed in CCA is attributed to membrane-bound β-Hp, not soluble β-Hp.

### 3.3 Detailed analysis of glycosylation structures of membrane-bound β-Hp

**Figure 3:**
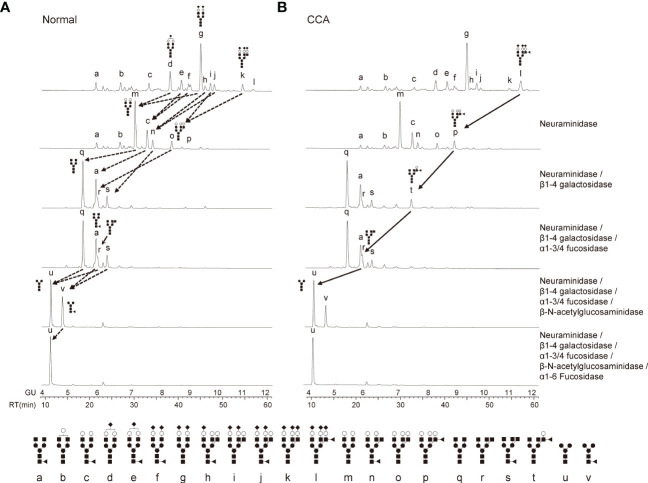
Detailed analysis of glycosylation structures in EV-Hp. **(A-B)** The chromatogram of 2-AB glycans extracted from EV-Hp of normal **(A)** and CCA **(B)**. Chromatograms of 2-AB glycans digested with various exoglycosidases are shown in lanes 2-6; n = 4. Glycan structures corresponding to peaks were shown below. ◼: GlcNAc, ●: mannose, ○: galactose, ▲: fucose, ◆: sialic acid.

To analyze the detailed glycosylation structures of EV-Hp, we conducted HILIC-HPLC and found that the glycans of EV-Hp from normal and CCA exhibited similar chromatograms with 12 major peaks (**Figures 3A, B**). The most abundant structure in both Hps was A2G2S2 (**Figures 3A, B**; peak “g”). Major differences between samples were tri-antennary glycans shown in the peak “k” and “l”. Afucosylated glycans were higher in normal than in CCA, whereas the opposite occurred for glycans having terminal fucosylation, which were higher in CCA than in normal (**Figures 3A, B**). The glycan structures were verified by treatment with exo-glycosidases. In particular, the peak “l” was shifted to peak “p” by neuraminidase and then to peaks “t” and “r” by sequential treatment with β1-4 galactosidase and α1-3/4 fucosidase (**Figure 3B**). However, core fucosylated glycans in the peak “v”, which disappeared by treatment with α1-6 fucosidase, did not show a significant difference between samples (**Figure 3B**). These results suggest that EV-Hp from CCA patients exhibits increased α1-3/4 fucosylated glycans (terminal fucosylation) compared to that from healthy controls.

### 3.4 Membrane-bound β-Hp with terminal glycosylation originated from CCA cells

**Figure 4:**
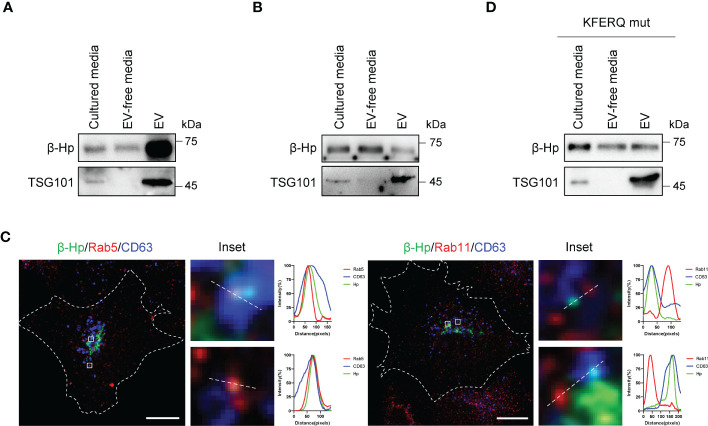
EV-Hp is secreted from CCA cells *via* KFERQ-like motif. **(A, B)** Two forms of Hp obtained from cultured media of **(A)** SNU-1079 and **(B)** HepG2 were examined; n = 5. **(C)** Colocalization of β-Hp with Rab5, Rab11, and CD63. Representative images are shown, Scale bar = 10 µm. n = 3. **(D)** Western blotting to assess the effects of KFERQ-like motif on β-Hp secretion through EV; n = 5.

We next sought to examine how β-Hp, previously known as a soluble secretory protein, could be detected in membrane-bound fraction. When β-Hp was expressed in SNU-1079, it was preferentially secreted through EV (**Figure 4A**). On the other hand, β-Hp expressed by HepG2 was secreted in a soluble from (**Figure 4B**). Additionally, real-time tracking of protein trafficking using RUSH system showed that newly synthesized β-Hp was secreted through the conventional secretory pathway in SNU-1079 cells (**Supplementary Figure 2A**). When β-Hp exited the Golgi, it was transported through Rab5-positive endosome, which co-localized with CD63, but not through Rab11-positive endosome (**Figure 4C**). We also noted that β-Hp possesses the phosphorylation-generated KFERQ-like motif required for cargoes to be sorted into EV (**Supplementary Figures 2B**) (25). When the motif was disrupted, β-Hp was secreted in a soluble form from SNU-1079 (**Figure 4D**).

**Figure 5:**
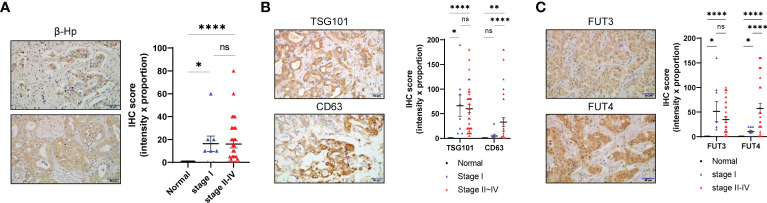
Fucosylation of EV-Hp is upregulated by CCA. **(A)** IHC staining of β-Hp in CCA tissue. Scale bar = 50 μm. IHC scores are shown. **(B)** IHC staining of TSG101 and CD63 in CCA tissue. Scale bar = 50 μm. IHC scores are shown. **(C)** IHC staining of FUT3 and FUT4 in CCA tissue. Scale bar = 50 μm. IHC score is shown. Quantitative data are shown as mean ± S.E.M. Statistical analysis was performed using the Mann-Whitney u-test. ****p < 0.0001, **p < 0.01, *p < 0.05, ns p > 0.05. ns, not significant.

We next investigated whether genes associated with EV-Hp fucosylation are upregulated in CCA. Evaluating GSE26566 dataset revealed that multiple genes involving glycosylation showed altered expression in CCA compared with the surrounding liver and normal intrahepatic bile duct (**Supplementary Figure 3A**). Among a family of FUT required for α1-3/4 fucosylation (34), FUT3 and FUT4 were particularly increased in CCA (**Supplementary Figures 3B, C**). We also found in OncoDB that MGAT5 required for β1-6GlcNAc side chain was upregulated in CCA (**Supplementary Figure 3C**). Our analysis using immunohistochemistry staining on 42 patients with CCA revealed high expression levels of β-Hp, TSG101, CD63, FUT3, and FUT4 (**Figures 5A–C**). Interestingly, FUT3 was highly expressed from early-stage cancer, whereas FUT4 gradually increased with tumor progression (**Figure 5C**). Collectively, our data suggest that terminal fucosylation of EV-Hp is regulated and secreted by CCA cells.

### 3.5 Clinical significance of fucosylated membrane-bound β-Hp in CCA diagnosis

**Figure 6:**
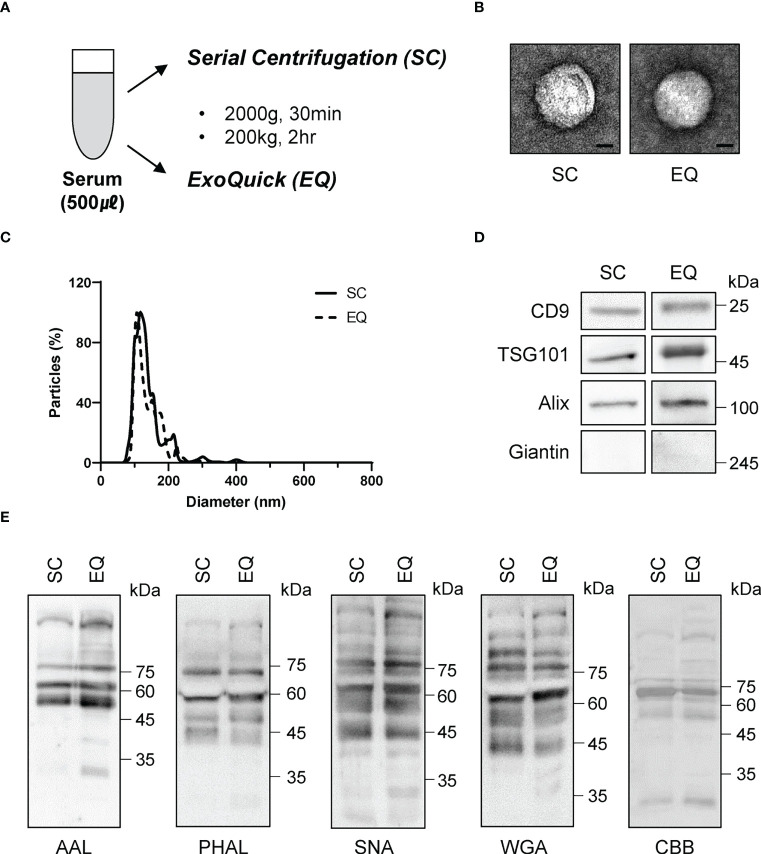
Comparison of glycosylation between EVs isolated *via* two different approaches. **(A)** Schematic of the EV isolation methods. SC, serial centrifugation. EQ, ExoQuick. **(B)** Electron microscopy (EM) assessment of the size and morphology of isolated EVs. Representative images are shown. Scale bar = 20 nm. **(C)** Nanoparticle tracking analysis for the quantitation of EV size. **(D)** Western blotting using antibodies against EV components. n = 3. **(E)** Lectin blot assessing the glycosylation profiles of isolated EVs. The blotted membrane was stained with Coomassie Brilliant Blue, n = 3.

To investigate the diagnostic value of fucosylated membrane-bound Hp, we first ensured that the isolation procedure did not alter the glycosylation of EVs. We obtained EVs from the serum using two different methods: serial centrifugation (SC) and Exoquick (EQ) (**Figure 6A**). We confirmed that the EVs obtained from both methods were spherical and had a diameter of approximately 100 nm using electron microscopy and nanoparticle tracking analysis (**Figures 6B, C**). We also detected similar levels of CD9, TSG101, and ALIX in both EVs, while the intracellular organelle component such as giantin was excluded (**Figure 6D**). Glycan analysis using various lectins confirmed that our isolation procedures did not affect the glycosylation status of EVs (**Figure 6E**).

**Figure 7:**
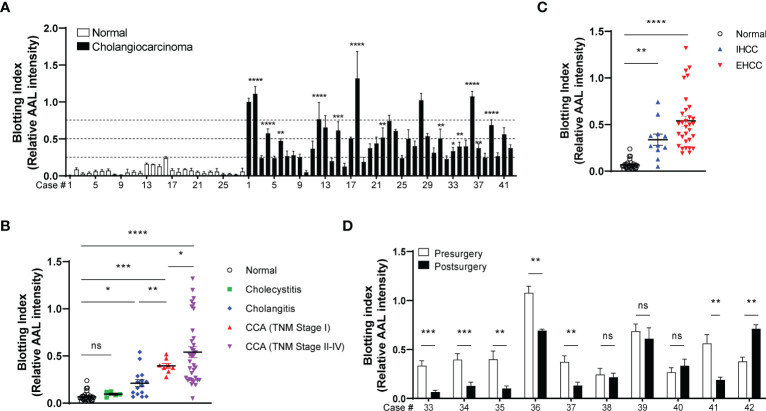
Diagnostic and prognostic value of EV-Hp fucosylation in CCA. Quantitative data are shown as mean ± S.E.M. Statistical analysis was performed using the two-tailed Student’s t-test. ****p < 0.0001, ***p < 0.001, **p < 0.01, *p < 0.05, ns, p > 0.05. **(A)** AAL blotting for EV-Hp of CCA and healthy volunteers. The fucosylation level was examined using a blotting index; n = 7. **(B)** Comparison of EV-Hp fucosylation for different stages of CCA, inflammatory conditions, and normal. **(C)** Effect of tumor location on EV-Hp fucosylation. IHCC, intrahepatic cholangiocarcinoma. EHCC, extrahepatic cholangiocarcinoma. **(D)** Effect of pre- and post-surgery to remove tumors on fucosylation of EV-Hp; n = 5. ns, not significant.

We next isolated EVs from the sera of patients with CCA (42 cases), inflammatory conditions (20 cases), and healthy volunteers (28 cases). AAL blotting revealed that the degree of EV-Hp fucosylation increased progressively with tumor progression (**Figures 7A, B** and **Table 1**). Fucosylation levels of cholecystitis and cholangitis were clearly distinct from that of the early-stage CCA patients (**Figure 7B**). We also observed that EV-Hp from EHCC was more fucosylated than that from IHCC (**Figure 7C** and **Table 1**). Next to examine the prognostic significance, EVs were obtained from patients with CCA before and after surgery to remove tumors. Six of the 10 cases showed a significant reduction in fucosylation, with a dramatic decrease in serum CA19-9 following the removal of the primary tumor (**Figure 7D** and **Supplementary Table 1**). Intriguingly, the AAL reactivity was not altered in four patients diagnosed with distant metastasis (CCA no. 38), tumor recurrence (CCA no. 39 and 40), and incomplete tumor removal (CCA no. 42) after surgery (**Figure 7D**). We further confirmed that fucosylation level of unfractionated serum was not correlated with prognostic factors (**Supplementary Figure 4**). These results suggest that fucosylated EV-Hp can be used not only for early diagnosis of CCA, but also for predicting variability following surgical intervention and tumor recurrence after surgery.

**Table 1:** Blotting index of CCA cases with tumor stage, location, sex, and age.

	Total	Blotting index (case no, %)		
		<0.25	0.25-0.5	>0.5
Normal	28	28(100%)	–	–
CCA	42	9(21.43%)	16(38.09%)	17(40.48%)
Clinical stage				
Stage I	8	–	7(88.89%)	1(11.11%)
Stage II~IV	34	9(26.47%)	9(26.47%)	16(47.06%)
Tumor location				
EHCC	31	6(19.35%)	10(32.26%)	15(48.39%)
IHCC	11	3(27.27%)	6(54.55%)	2(10.18%)
Sex				
Male	29	7(24.14%)	12(41.38%)	10(34.48%)
Female	13	2(15.39%)	4(30.77%)	7(53.84%)
Age				
40~50	1	–	–	1(100%)
50~60	6	2(33.33%)	2(33.33%)	2(33.33%)
60~70	13	4(30.77%)	7(53.85%)	2(15.38%)
70~80	20	3(15%)	6(30%)	11(55%)
80~90	2	–	1(50%)	1(50%)

CCA, cholangiocarcinoma; EHCC, extrahepatic cholangiocarcinoma; IHCC, intrahepatic cholangiocarcinoma.

### 3.6 Fucosylated EV promotes the growth of CCA cells

**Figure 8:**
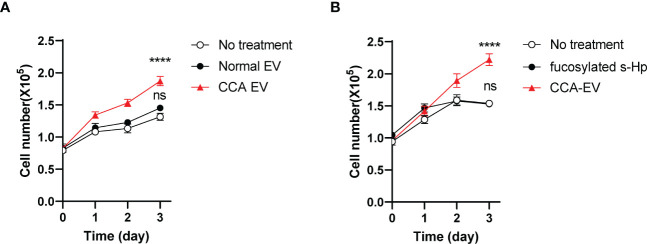
Fucosylated EV promotes CCA progression. Quantitative data are shown as mean ± S.E.M. Statistical analysis was performed using the two-tailed Student’s t-test: ****p<0.0001, ns p>0.05. **(A)** The effect of CCA EVs on the growth of SNU-1079 cells, n=3. **(B)** The impact of fucosylated soluble-Hp on cell growth, n=3. ns, not significant.

We next investigated the impact of fucosylated EV-Hp on tumor progression. The results showed that treatment with fucosylated EVs isolated from patients with CCA led to a significant increase in the growth of SNU-1079 cells (**Figure 8A**). However, treatment with the same amount of EVs isolated from healthy controls did not affect the growth of CCA cells (**Figure 8A**). Moreover, we demonstrated that the treatment of the soluble form of fucosylated Hp isolated from the serum of CCA patients did not influence cell growth, further emphasizing the importance of the EV-associated Hp (**Figure 8B**). Collectively, these findings suggest that fucosylated EVs derived from CCA contribute to tumor progression.

## 4 Discussion

Tumor glycosylation has been proposed as potential cancer biomarkers (6, 7). Many studies have focused on characterizing the aberrant glycosylation of secreted molecules circulating the blood in their soluble form (35). In this study, we provide new evidence that terminal fucosylation of membrane-bound components correlated with EVs correlates with CCA. The fucosylation was progressively increased with tumor stage, but was not increased in inflammatory conditions. Notably, EV fucosylation was significantly reduced when the tumors were removed by surgery.

Glycomic analysis revealed that CCA-EV contained more α1-3/4 fucosylated glycans (terminal fucosylation) than normal EVs. Notably however, α1-6 fucose (core fucosylation) was comparable between samples, and bisected fucosylated structures reported to increase in tissues of intrahepatic CCA could not be found (36). Our current approach did not distinguish the linkage of terminal α-L-fucose attached to tri-antennary structures. However, we pursued to explain it by evaluating the expression of different FUTs. For example, FUT3 is involved in type I Lewis antigens (Galβ1-3GlcNAc-R) with α1-4 fucosylation, whereas FUT4 is required for the synthesis of type II Lewis antigens (Galβ1-4GlcNAc-R) with α1-3 fucosylation (37–39). Our data showed that FUT3 was upregulated from the early stage, while FUT4 progressively increased with the tumor stage, suggesting a linkage conversion of α-fucosylation as the tumor progresses. Since expression of N-acetylglucosaminyltransferase V (GnT-V) increases during caner development (40, 41), α1-3/4 fucose is often attached to β1-6GlcNAc branched structure. Consistent with previous findings, we found that α1-3/4 fucosylation was detected only in tri-antennary structures and MGAT5 expression was upregulated in CCA.

Terminal fucosylation of CCA-EV was primarily attributed to the membrane-bound β-Hp. Our data uncovered that the glycosylation of soluble β-Hp was significantly different from EV-Hp. Remarkably, only the fucosylation of EV-Hp showed positive correlation with CCA. The key question raised how the two forms of β-Hp present differential glycosylation. As glycans are conjugated to the four N-linked glycosylation sites that are found in β-Hp (ASN-184, ASN-207, ASN-211, and ASN-241), site-specific glycan analysis will determine how the two forms of β-Hp differs. We also noted that β-Hp is primarily produced in hepatocytes and a small fraction is secreted by cancer cells (14, 15). One intriguing possibility is that the trafficking pathway through which β-Hp is secreted differs cell-to-cell. Our data uncovered that β-Hp expressed in CCA cells was secreted through EV, whereas β-Hp expressed in HepG2 was secreted in a soluble form. Considering that genes encoding β-Hp, EV biogenesis, and terminal fucosylation were upregulated in CCA cells, we conclude that fucosylated membrane-bound β-Hp originates from CCA.

In summary, our data uncovered that the α1-3/4 fucosylation of β-Hp in cancer-derived EV can be used as biomarkers for early diagnosis of CCA as well as for the prediction of recurrence after surgery. We also elucidated the molecular mechanisms of how CCA-specific glycosylation is regulated. Future research should further investigate how altered glycosylation involves EV biogenesis and the physiological relevance of EV fucosylation in cancer progression.

## Data availability statement

The original contributions presented in the study are included in the article/**Supplementary Material**. Further inquiries can be directed to the corresponding authors.

## Ethics statement

This study was approved by the Institutional Review Board of Chungnam National University Hospital (IRB file no. CNUH 2020-09-015). The patients/participants provided their written informed consent to participate in this study.

## Author contributions

Conceptualization: HC, S-YP. Methodology: HC, SJ, KK, M-HS, M-KY, S-YP. Validation: HC, SJ, M-KY, S-YP. Formal analysis: HC, S-YP. Investigation: HC, SJ, KK, M-HS. Resources: J-MK, M-KY. Writing - original draft: S-YP. Writing - review and editing: HC, SJ, EM, M-KY, S-YP. Visualization: HC. Supervision: S-YP. Project administration: S-YP. Funding acquisition: S-YP. All authors contributed to the article and approved the submitted version.
