# Supplementary material for: Terminal fucosylation of haptoglobin in cancer-derived exosomes during cholangiocarcinoma progression
Source: Front Oncol. 2023 Apr 24;13:1183442. doi: 10.3389/fonc.2023.1183442 (PMC10165115; doi:10.3389/fonc.2023.1183442)
Supplement: Supplementary file 1 [file DataSheet_1.pdf]

Supplementary Material

Terminal fucosylation of haptoglobin in cancer-derived exosomes during cholangiocarcinoma progression

Hyewon Choi[1], Sungeun Ju[1], Keunsoo Kang[2], Moon-Hyeong Seo[3], Jin-Man Kim[4], Eiji-Miyoshi[5], Min-Kyung Yeo[4,*], Seung-Yeol Park[1,*]

[1] Department of Life Sciences, Pohang University of Science and Technology (POSTECH), Pohang, Gyeongbuk, Republic of Korea

[2] Department of Microbiology, Dankook University, Cheonan, Chungnam, Republic of Korea

[3] Natural Product Research Center, Korea Institute of Science and Technology, Gangneung, Republic of Korea

[4] Department of Pathology, Chungnam National University School of Medicine, Daejeon, Republic of Korea

[5] Department of Molecular Biochemistry and Clinical Investigation, Osaka University Graduate School of Medicine, 1-7 Yamada-oka, Suita, Osaka, Japan

*** Correspondence:**
Professor. M.-K.Y. (Department of Pathology, Chungnam National University School of Medicine, Munwha-ro 282, Daejeon 35015, Republic of Korea, phone: +82-42-280-7196, e-mail: mkyeo83@cnu.ac.kr)

Professor. S.-Y.P. (Department of Life Sciences, POSTECH, Chungam-ro 77, Pohang, Gyeongbuk 37673, Republic of Korea, phone: +82-54-279-2325, e-mail: seungpark@postech.ac.kr)

1 Supplementary Figures and Tables

1.1 Supplementary Figure 1

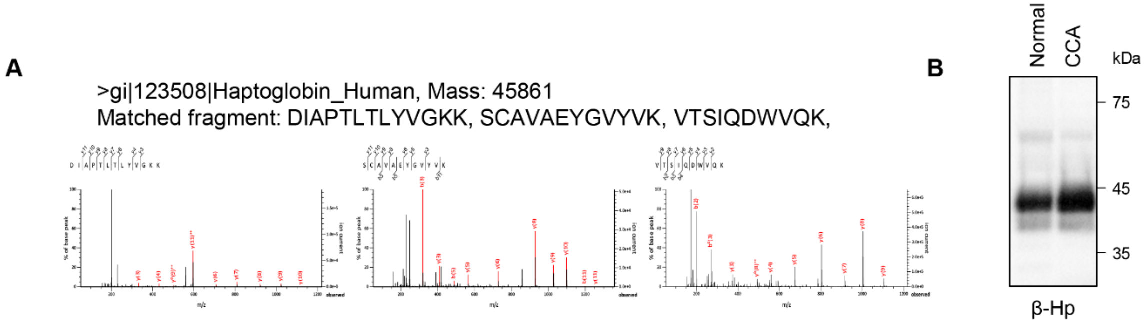

Supplementary Figure 1. Identification of 40kDa protein with aberrant fucosylation in CCA. (A) Mass spectrum of 40kDa protein. The identified peptides matched the sequence of β-Hp. (**B)** Western blot assessing the amount of β-Hp in membrane-bound fraction isolated from healthy volunteers and patients with CCA; n = 3.

1.2 Supplementary Figure 2

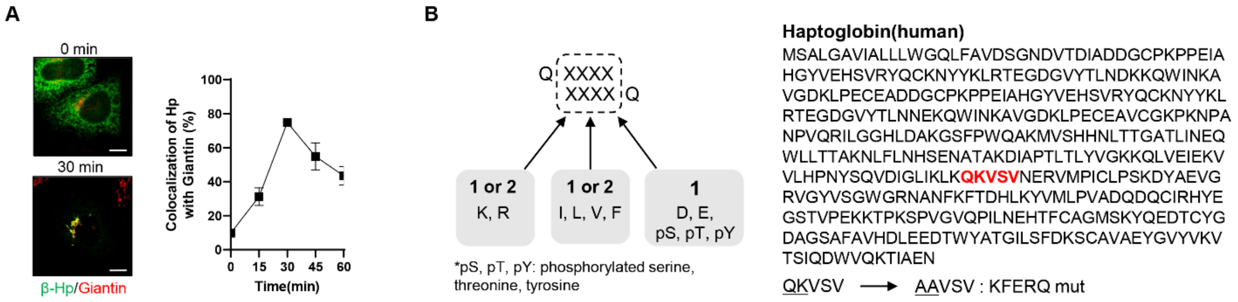

Supplementary Figure 2. Further characterization of β-Hp secreted through EV. (A) RUSH assay to assess the real-time tracking of β-Hp secretion. Representative confocal images are shown, Scale bar = 10 μm. n = 3. (B) (left) Scheme of canonical and phosphorylation-generated KFERQ-like motifs. (right) KFERQ-like motif in haptoglobin.

1.3 Supplementary Figure 3

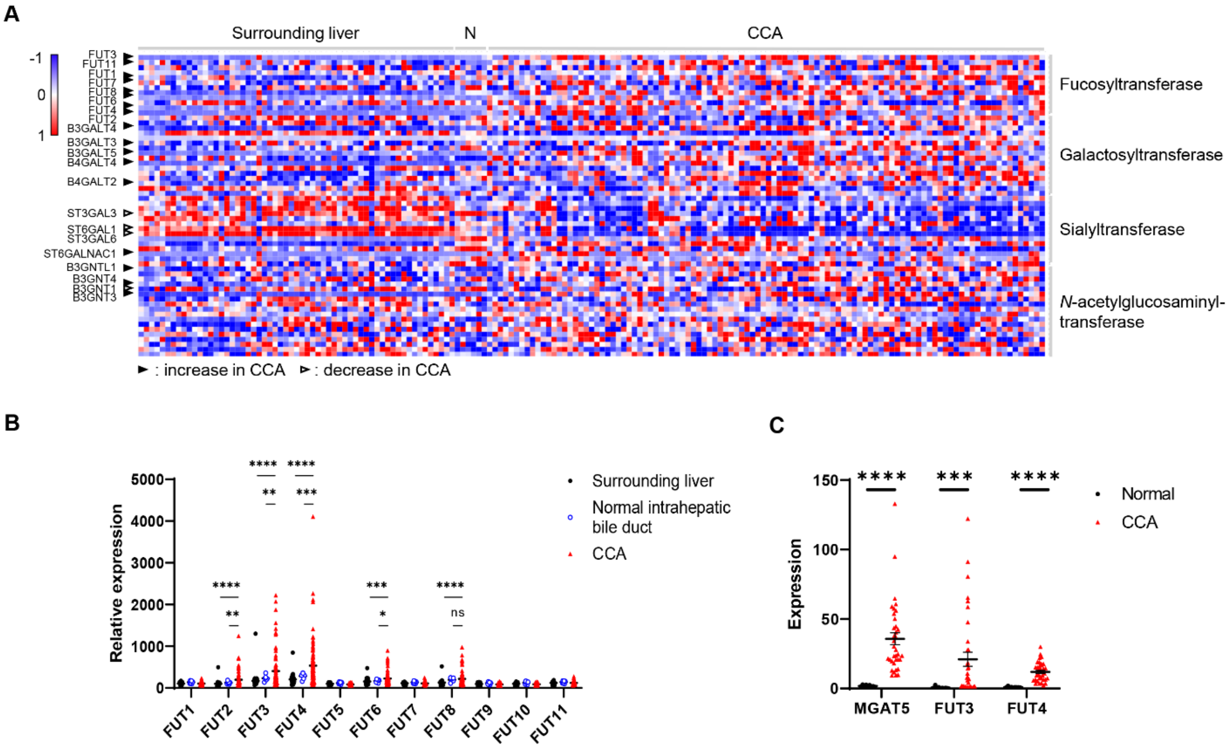

Supplementary Figure 3. Expression profile of genes involving glycosylation biosynthesis. Quantitative data are shown as mean ± S.E.M. Statistical analysis was performed using the Mann-Whitney u-test: ****p < 0.0001, ***p < 0.001, **p < 0.01, *p < 0.05, ns P > 0.05. (A) The heatmap represents the expression level (log 2-fold change) of genes in the surrounding liver, normal intrahepatic bile duct (N), and CCA. (B) Expression profiles of fucosyltransferases (FUTs) required for α-fucosylation. (C) Expression profiles of MGAT5, FUT3, and FUT4 evaluated in OncoDB.

1.4 Supplementary Figure 4

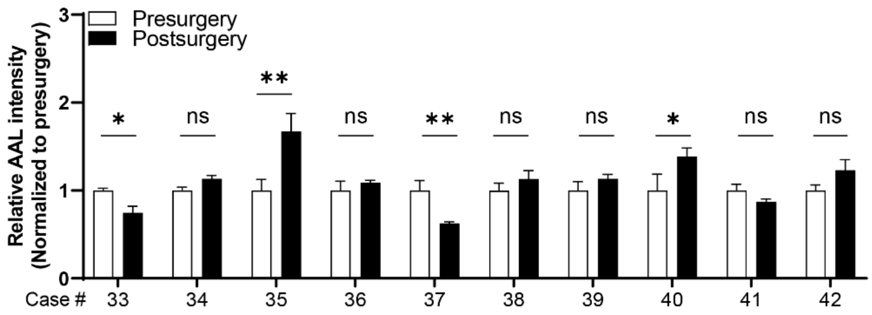

Supplementary Figure 4. Effect of pre- and post-surgery on fucosylation degree of unfractionated serum. Quantitative data are shown as mean ± S.E.M. Statistical analysis was performed using the two-tailed Student's t-test: **p < 0.01, *p < 0.05, ns P > 0.05. AAL blotting for unfractionated serum collected from CCA patients before and after the surgery to remove tumors. Fucosylation level was determined using blotting index; n = 5.

1.5 Supplementary Table 1

Supplementary Table 1. Clinical information of CCA cases. F: female, M: male, EHCC: extrahepatic cholangiocarcinoma, IHCC: intrahepatic cholangiocarcinoma.

Case No.	Age	Sex	Location	Tstage	Nstage	Mstage	TNM stage (AJCC)
1	77	F	EHBD	T1	N1	M0	IIA
2	42	F	EHBD	T3b	N1	M0	IIIA
3	51	M	EHBD	T2	N1	M0	IIB
4	70	M	EHBD	T2a	N0	M1	IVB
5	53	M	EHBD	T3a	N1	M0	IIIA
6	82	F	EHBD	T1	N0	M0	I
7	78	M	EHBD	T2	N0	M0	IIA
8	53	M	IHBD	T1a	N0	M0	IA
9	62	F	IHBD	T2	N0	M0	II
10	77	F	IHBD	T2	N0	M0	II
11	69	F	EHBD	T3	N0	M0	IIB
12	56	M	EHBD	T2	N0	M0	IIA
13	71	F	EHBD	T2	N0	M0	IIA
14	61	M	EHBD	T2	N0	M0	IIA
15	76	M	EHBD	T2	N1	M0	IIB
16	69	M	IHBD	T3	N0	M0	IIIA

17	73	M	EHBD	T3	N0	M0	IIB	
18	69	M	EHBD	T2	N0	M0	IIA	
19	72	M	EHBD	T2	N0	M0	IIA	
20	61	M	IHBD	T1a	N0	M0	IA	
21	77	M	EHBD	T3	N0	M0	IIB	
22	73	F	EHBD	T1	N0	M0	I	
23	77	M	IHBD	T1a	N0	M0	II	
24	73	F	IHBD	T1a	N0	M0	II	
25	64	M	EHBD	T2	N1	M0	IIB	
26	69	M	EHBD	T2b	N0	M0	II	
27	70	M	EHBD	T3a	N1	M0	IIIA	
28	74	M	EHBD	T2	N0	M0	IIA	
29	77	F	EHBD	T2a	N0	M0	II	
30	70	M	EHBD	T3	N0	M0	IIB	
31	56	M	EHBD	T2b	N0	M0	II	
32	75	M	IHBD	T2	N0	M0	II	
**Serum collected before and after surgery**								
**Case No.**	**Age**	**Sex**	**Location**	**Tstage**	**Nstage**	**Mstage**	**TNM stage (AJCC)**	**Postsurgery CA19-9 (unit)**
33	69	M	EHBD	T1	N0	M0	I	16
34	64	M	IHBD	T1a	N0	M0	IA	5.64
35	74	M	EHBD	T1	N0	M0	I	8.24
36	84	M	EHBD	T2a	N0	M0	II	2.78
37	56	M	EHBD	T2	N0	M0	IB	16.1

38	66	F	EHBD	T4	N1	M0	IIIB	362.1
39	65	M	EHBD	T2b	N0	M0	II	2516
40	65	F	IHBD	T3	N1	M0	IIIB	4262
41	79	F	EHBD	T2	N0	M0	IIA	4.87
42	75	M	IHBD	T2	N0	M0	II	21.74
